# Intermittent Sequential Pneumatic Compression Improves Coupling between Cerebral Oxyhaemoglobin and Arterial Blood Pressure in Patients with Cerebral Infarction

**DOI:** 10.3390/biology10090869

**Published:** 2021-09-04

**Authors:** Wenhao Li, Gongcheng Xu, Congcong Huo, Hui Xie, Zeping Lv, Haihong Zhao, Zengyong Li

**Affiliations:** 1 Key Laboratory for Biomechanics and Mechanobiology of Ministry of Education, School of Biological Science and Medical Engineering, Beihang University, Beijing 100191, China; wenhaoli@buaa.edu.cn (W.L.); gongchengxu93@buaa.edu.cn (G.X.); congconghuo@buaa.edu.cn (C.H.); xiehui@buaa.edu.cn (H.X.); 2 Beijing Advanced Innovation Center for Biomedical Engineering, Beihang University, Beijing 100083, China; 3 Rehabilitation Hospital, National Research Center for Rehabilitation Technical Aids, Beijing 100176, China; Lvzeping@nrcrta.cn (Z.L.); zhaohaihongqian@163.com (H.Z.); 4 Beijing Key Laboratory of Rehabilitation Technical Aids for Old-Age Disability, National Research Center for Rehabilitation Technical Aids, Beijing 100176, China; 5 Key Laboratory of Neuro-Functional Information and Rehabilitation Engineering of the Ministry of Civil Affairs, Beijing 100176, China

**Keywords:** intermittent sequential pneumatic compression, arterial blood pressure, cerebral hemodynamic, dynamic Bayesian inference

## Abstract

**Simple Summary:**

Cerebral autoregulation is a homeostatic feedback mechanism that maintains a relatively constant cerebral blood flow despite changes in blood pressure. Impaired cerebral autoregulation results in unstable cerebral blood flow and is detrimental to the outcome of neurological diseases. Therefore, metrics of cerebral autoregulation were increasingly used to assess cerebrovascular health or to guide hemodynamic management. Intermittent sequential pneumatic compression (ISPC) is an effective physiotherapy technique that could improve motor deficits in patients with acute cerebral infarction. The present study focuses on the coupling relationships between arterial blood pressure and changes in oxygenated hemoglobin in the cerebral cortex to investigate the effect of ISPC on cerebral autoregulation in patients with cerebral infarction, as compared with the healthy controls. This study may help to advance the understanding of the physiological mechanisms of ISPC intervention and can provide a basis for evaluating the efficacy of ISPC interventions in patients with cerebral infarction.

**Abstract:**

This study aims to explore the effect of intermittent sequential pneumatic compression (ISPC) intervention on the coupling relationship between arterial blood pressure (ABP) and changes in oxyhaemoglobin (Δ [O_2_Hb]). The coupling strength between the two physiological systems was estimated using a coupling function based on dynamic Bayesian inference. The participants were 22 cerebral infarction patients and 20 age- and sex-matched healthy controls. Compared with resting state, the coupling strength from ABP to Δ [O_2_Hb] oscillations was significantly lower in the bilateral prefrontal cortex (PFC), sensorimotor cortex (SMC), and temporal lobe cortex (TLC) during the ISPC intervention in cerebral infarction patients in interval II. Additionally, the coupling strength was significantly lower in the bilateral SMC in both groups in interval III. These findings indicate that ISPC intervention may facilitate cerebral circulation in the bilateral PFC, SMC, and TLC in cerebral infarction patients. ISPC may promote motor function recovery through its positive influences on motor-related networks. Furthermore, the coupling between Δ [O_2_Hb] and ABP allows non-invasive assessments of autoregulatory function to quantitatively assess the effect of rehabilitation tasks and to guide therapy in clinical situations.

## 1. Introduction

Stroke is a leading cause of disability worldwide, resulting in a substantial burden on caregivers and society [1]. Survivors experience the deterioration or loss of functions, such as sensorimotor sequelae, cognitive deficits, and psychiatric deficits. Research has demonstrated that the dysfunction of the motor system following stroke might be related to damage to the brain motor-related cortex [2]. Early and effective rehabilitation can improve the plasticity of the central nervous system, maximizing the recovery of impaired motor function, reducing the disability level, and improving the prognosis. It is therefore of great significance to evaluate the effectiveness of rehabilitation interventions in patients who have experienced a stroke.

Recent evidence suggests that a further insight of the regulatory mechanism of the brain–cardiovascular system might be obtained through research on both cerebral hemodynamics and other cardiovascular parameters [3]. Cerebral autoregulation is an important physiological marker in cerebrovascular diseases that describes the regulatory response of cerebral hemodynamic variables to changes in blood pressure [4]. Impaired cerebral autoregulation results in unstable cerebral blood flow and is detrimental to the outcome of neurological diseases. Currently, an increasing number of researchers are using metrics of cerebral autoregulation to assess cerebrovascular health or to guide hemodynamic management. Intermittent sequential pneumatic compression (ISPC) is an effective physiotherapy technique that could improve motor function defects in patients with cerebral infarction and motor impairment [5]. Combining this technique with rehabilitation training can effectively ameliorate neurological deficits and restore a certain degree of motor function to the limbs of patients who have experienced cerebral infarction [5]. Previous study has suggested that the application of ISPC will lead to an increase in hemodynamic shear stress, which can induce the improvement of endothelial function and growth of collateral arteries [6]. However, brain—cardiovascular interactions during ISPC intervention are not well understood.

The introduction of functional near-infrared spectroscopy (fNIRS) provides a powerful tool to assess cerebral autoregulation noninvasively. fNIRS is an emerging non-invasive neuroimaging technique that enables the investigation of brain hemodynamic with reasonable temporal and spatial resolution, which can quantify the task-related changes in oxygenated haemoglobin (Δ [O_2_Hb]) and deoxygenated haemoglobin (Δ [HHb]) concentrations [7,8]. This method has several advantages including reasonable temporal and spatial resolution, portability, and relatively low susceptibility to motion artifacts [9]. These characteristics provide fNIRS with substantial advantages in investigating cerebral activity in stroke rehabilitation. Continuous wavelet transform is a powerful mathematical tool for analyzing stationary and nonstationary time series in the time-frequency domain [10]. Based on continuous wavelet transform, several studies have investigated the relationship between arterial blood pressure (ABP) and fNIRS-measured parameters in the study of cerebral autoregulation [10,11]. The wavelet parameters and derived metrics include wavelet coherence, wavelet phase coherence, and wavelet cross-correlation [12]. However, none of these methods provide information about causality or the form of the coupling functions [13]. With the development of nonlinear dynamics, a coupling function based on dynamic Bayesian inference analysis technology was used to assess cerebral autoregulation function [14]. This method could extract and reconstruct the coupling functions between interacting oscillations from data [3]. The influence of the unidirectional driving that one oscillator exerts on the other could be quantitatively described by calculating the coupling strength [15].

It has previously been observed that the activation of the bilateral prefrontal and parietal cortex might be involved in the recovery process after stroke [16]. Additionally, motor recovery is related to activation in sensorimotor regions in patients with hemiplegic stroke [17]. We speculated that the application of ISPC would affect the coupling strength between ABP and Δ [O_2_Hb] in the prefrontal cortex (PFC), sensorimotor cortex (SMC), and temporal lobe cortex (TLC). In this study, we used a 24-channel fNIRS instrument and a noninvasive blood pressure device to measure the dynamic changes in [O_2_Hb] and ABP, respectively, at rest and undergoing ISPC intervention. The phase was extracted from the measured variables using the continuous wavelet transform. Then, the coupling between ABP and the fNIRS-measured quantities was calculated from the coupling function based on the dynamic Bayesian inference technique. These results may facilitate the advancement of the comprehension of the physiological mechanisms of ISPC intervention and can provide a basis for evaluating the efficacy of ISPC interventions in patients with cerebral infarction.

## 2. Materials and Methods

### 2.1. Participants

A total of 22 cerebral infarction patients (Group Stroke) and 20 age- and sex-matched healthy participants (Group Control) were recruited in this study. The demographic characteristics of all participants are summarized in Table 1. Each participant was fully informed of the project information including the research procedures, potential benefits, and risks. Written informed consent was obtained from the patient or their legally authorized representative before the study. The study was conducted in accordance with the Declaration of Helsinki, and the protocol was approved by the Human Ethics Committee of Rehabilitation Hospital, National Research Center for Rehabilitation Technical Aids (Project 20180109).

None of the healthy participants had a history of cardiovascular or peripheral vascular disease and were not taking any medication known to affect neurological function. Patients who had experienced cerebral infarction were recruited through the inpatient Department of Rehabilitation Hospital, National Research Center for Rehabilitation Technical Aids, China. The inclusion criteria were: (1) right-handed; (2) within 6 months after the onset of stroke; (3) unilateral hemiplegia and moderate to severe motor deficits of the upper and lower extremities on the hemiplegic side; and (4) stable condition after stroke without neurofunctional disabilities. The exclusion criteria were: (1) patients with varicose veins; (2) patients with local leg problems in the area where the ISPC sleeves would be applied, such as dermatitis or open wounds on the legs; (3) patients with severe atherosclerosis as indicated by an absence of pedal pulses or a history of definite intermittent claudication; and (4) patients with severe leg edema or pulmonary edema from congestive heart failure. Table 2 presents the individual characteristics of the cerebral infarction patients.

### 2.2. Instrumentation

**Figure 1:**
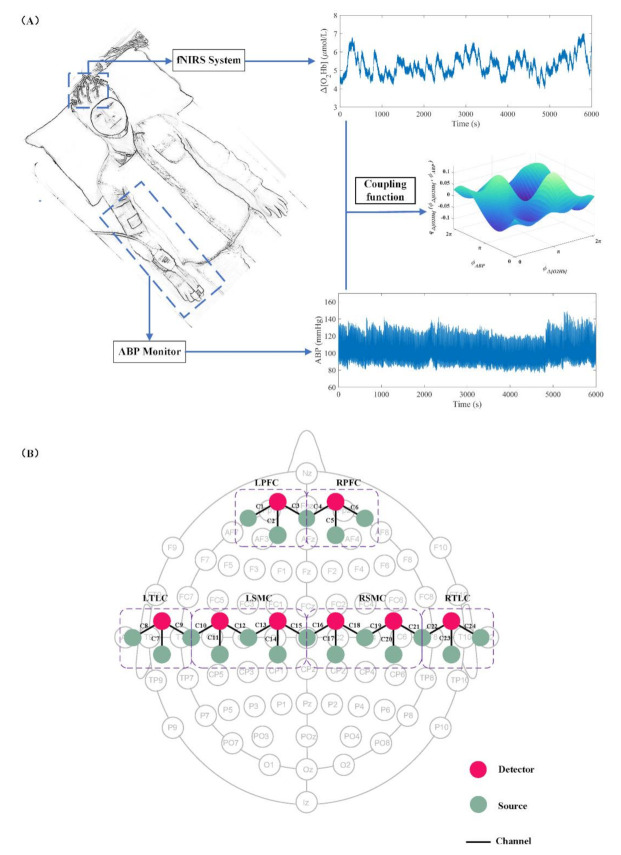
(**A**) Schematic of the experimental layout. (**B**) Schematic of the channel configuration of the probes. Probe geometry with 18 light sources (green) and 8 detectors (red). The ‘C’ means channel. Six cerebral cortex areas (LPFC, RPFC, LSMC, RSMC, LTLC, and RTLC) were separated by the rectangular.

The hemodynamic data (Δ [O_2_Hb] and Δ [HHb]) were monitored with a multichannel fNIRS system (NirSmart, Danyang Huichuang Medical Equipment CO. Ltd, Danyang, China) at two wavelengths (760 and 850 nm). The raw sampling frequency was set to 10 Hz. Eighteen light sources and eight detectors interlacing at a spatial distance of 3.0 cm were plugged into a soft but inelastic plastic probe holder and placed on the head of the participants. These source-detector pairs constituted 24 measurement channels that were symmetrically distributed on the bilateral hemispheres and covered cerebral regions of the PFC, SMC, and TLC. The probes were positioned according to the international 10/10 system. The channel configuration of the probes is shown in Figure 1. To improve the signal-to-noise ratio, the hair under each probe was manually parted prior to the experiment. To facilitate the presentation of the results, the data of the left and right sides of the brain of patients with right hemiplegia were replaced at the channel level. Therefore, the left hemisphere of the brain represents the contralesional (CL) region and the right hemisphere of the brain represents the ipsilesional (IL) region in the following sections of this paper.

Simultaneous with the fNIRS signals, continuous ABP signals were measured by a noninvasive blood pressure device (CNAP^TM^ Monitor 500, CNSystems Medizintechnik AG, Austria) at a sampling frequency of 2000 Hz. The ABP signal was acquired by the software AcqKnowledge (BIOPAC Systems Inc., Goleta California, USA).

The ISPC device (DSM-800S, Daesung Maref Co., Ltd, gunpo-si, Korea) consisted of a pneumatic pump, inflatable auxiliary sleeves, and pressure lines. The inflatable auxiliary sleeves were wrapped around the upper and lower extremities on the hemiparetic side and secured by Velcro. The sleeves were connected to the air pump via pressure lines. The inflation pressure range of the ISPC device was 10–200 mmHg.

### 2.3. Experimental Design

All measurements were performed with the participants resting in the supine position. Prior to the experiment, each participant was required to rest in a quiet environment for 5 min to eliminate the existing hemodynamic reactions induced by previous activity. Each participant then underwent a 10-minute resting-state session. During this stage, participants were instructed to close their eyes and relax, while remaining awake and as still as possible. Subsequently, ISPC intervention (20 min) was performed on the hemiplegic limb for Group Stroke and on the left limb for the Group Control. The 10-minute resting-state session was defined as State_R, and the data from 3 min to 18 min in the ISPC rehabilitation training state were defined as State_ISPC. One full compression from distal to proximal is called a cycle. The compression rate of the ISPC device was 2 cycles/min (15 s inflation/15 s deflation). The clinical pressure range used was 100–150 mmHg. To ensure patient safety, the inflation pressure was adjusted mainly according to the patient’s comfort and tolerance. All operations were performed by a professional therapist.

### 2.4. Data Preprocessing and Spectral Analysis

The Δ [HHb] signal may be less contaminated by global processes compared with the Δ [O_2_Hb] [18]. Therefore, only the Δ [HbO_2_] signal was analyzed in the following study. To measure the coupling between the Δ [O_2_Hb] and ABP signals, it is necessary to extract the information characteristics representing the measured data.

#### 2.4.1. Data Preprocessing

With the modified Beer–Lambert law, the filtered optical density signals were converted to Δ [O_2_Hb] [19]. Then, for each participant, principal component analysis was applied to the Δ [O_2_Hb] signals to reduce the dimensionality of the hemoglobin data. Subsequently, independent component analysis was used on the reduced dimensional data to identify typical noise components, removing the technical and physiological artifacts, including nonspecific activity of the superficial layers [20]. The procedures of independent component analysis adopted in the present work were consistent with those of Zhang et al. (2010) [21], and independent component analysis was conducted with the Fast ICA v2.5 algorithm [22]. Finally, the moving average and cubic spline interpolation methods were adopted to eliminate noise-like abrupt spikes and motion artifacts, respectively [23]. The window width of the moving average method was 5 s. To achieve a uniform time basis, the raw ABP signal was downsampled to 10 Hz. The mean arterial blood pressure and heart rate were calculated by AcqKnowledge.

#### 2.4.2. Phase Extraction and Coupling Analysis

Continuous wavelet transforms were employed to extract the frequency range into three physiological bands of interest as previously defined from Δ [O_2_Hb] [24,25]: interval I, 0.2–0.5 Hz; interval II, 0.07–0.2 Hz; and interval III, 0.02–0.07 Hz. The spontaneous oscillations in interval I are related to the effects of respiratory activities [26]. The spontaneous oscillations in interval II are associated with vasomotion and sympathetic activity [4]. The spontaneous oscillations in interval III mainly reflect hemodynamic fluctuations that originate from spontaneous cortical neural activity [27].

**Figure 2:**
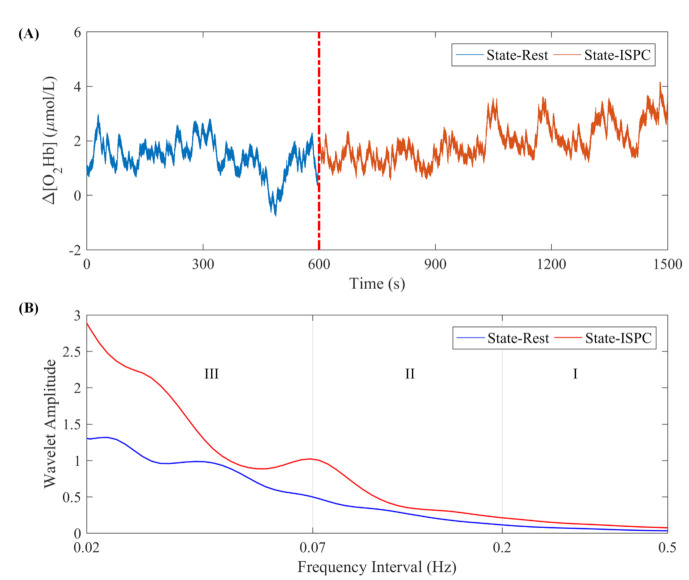
(**A**) Representative time series of the Δ [O_2_Hb] signal from one subject in State_R and State_ISPC. The red dotted line indicates the start of the ISPC intervention. (**B**) The corresponding wavelet amplitude of Δ [O_2_Hb] signal. The vertical lines indicate the outer limits of the frequency intervals: I, 0.2–0.5 Hz; II, 0.07–0.2 Hz; and III, 0.02–0.07 Hz.

The oscillatory component in a signal can be characterized by its instantaneous frequency and corresponding amplitude [28]. The wavelet amplitude is the average result of the continuous wavelet transforms in the time domain, which characterizes the amplitude of fluctuations in the original signal within a given frequency interval. Thus, the wavelet amplitude of the Δ [O_2_Hb] signal can be used to reflect the intensity of activity in cerebral regions [29]. A representative oscillation of Δ [O_2_Hb] during ISPC is shown in Figure 2.

The coupling function between ABP and Δ [O_2_Hb] signals is established based on dynamic Bayesian inference, which has previously been explained in detail [14]. The coupling function can characterize the interaction mechanism between oscillators. The specific effects of each oscillator on the others can be obtained by decomposing the coupling function into a set of interacting phase oscillators [15]. Following the determination of the phase of each time series with the continuous wavelet transform, their dynamics are assumed to be described by [30]:

(1) $${\dot{\psi }}_{i}\left(t\right)=w_{i}\left(t\right)+q_{i}\left(\psi_{i},\psi_{j},\psi_{k},\cdots ,\psi_{N},t\right)+\zeta \left(t\right)$$

with *i* = 1…*N*, where ${\dot{\psi }}_{i}\left(t\right)$ is the time derivative of the phase, $w_{i}\left(t\right)$ is the natural frequency, and $\zeta \left(t\right)$ is the Gaussian white noise. The deterministic periodic part $q_{i}$ can be Fourier-decomposed into a sum of base functions $\Psi_{k}=\exp\left[\iota \left(k_{1}\psi_{1}+k_{2}\psi_{2}+\cdots +k_{N}\psi_{N}\right)\right]$ [30], characterized by the time-varying bank of parameters $c_{k}^{\left(i\right)}$:

(2) $${\dot{\psi }}_{i}\left(t\right)=\overset{K}{\underset{k=-K}{\sum }}c_{k}^{\left(i\right)}\Psi_{k}\left(\psi_{1},\psi_{2},\cdots ,\psi_{n},t\right)+\zeta_{i}\left(t\right)$$

Second-order Fourier expansion (K = 2) is used in the analysis. Starting from the phase dynamics extracted from the time series, the aim is to compute the set of parameters $ℳ=\left\{c_{k}^{\left(i\right)},D_{r,s}\right\}$ that completely describes the couplings $c_{k}^{\left(i\right)}$ and the noise $D_{r,s}$.

Then, dynamic Bayesian inference is used to establish the coupling function between ABP and Δ [O_2_Hb] in different cerebral cortex regions. It allows one to obtain the posterior density $p_{\chi }\left(ℳ|X\right)$ of the unknown matrix of parameters ℳ from X, given a prior density $p_{prior}\left(ℳ\right)$, by building a likelihood function $\ell \left(X|ℳ\right)$ [31]:

(3) $$p_{\chi }\left(ℳ|X\right)=\frac{\ell \left(X|ℳ\right)p_{prior}\left(ℳ\right)}{\int \ell \left(X|ℳ\right)p_{prior}\left(ℳ\right)dℳ}$$

In this study, the quantified values of the coupling strengths are applied to measure the combined relationships between the oscillators. The coupling strength $CS_{i,j}$ from oscillator i to oscillator j is [13]:

(4) $$CS_{i,j}={\left(\underset{k}{\sum }{\left(c_{k}^{\left(i:j\right)}\right)}^{2}\right)}^{\frac{1}{2}}$$

All parameters are defined as in Equation (1).

### 2.5. Statistical Analysis

Both the Mann–Whitney and the Kolmogorov–Smirnov tests are nonparametric tests to compare two unpaired groups of data. But they work very differently. The Mann–Whitney test is mostly sensitive to changes in the median. By contrast, the Kolmogorov–Smirnov test is sensitive to differences in both location and shape. Substantial differences in shape, spread, or median will result in a small *p* value [1]. Therefore, in this study, the Kolmogorov–Smirnov test was selected. The normal test (Kolmogorov–Smirnov test) and variance uniformity test (Levene test) of each participant’s data were performed at the group level to ensure that the assumptions required for parameter analysis were satisfied. Two-way ANOVA was adopted for the coupling strength values to check whether significant interactions were present between stroke and the task factors (main effect of group and state and interaction of group × state). The significant differences in the changes in the wavelet amplitude values and coupling strength values with stroke and task were calculated with one-way ANOVA. In each group, two states for wavelet amplitude and coupling strength value comparison were designed (State_R vs State_ISPC, three intervals). In total, there were 3 × 2 = 6 (interval × condition) intergroup pairwise comparisons (two states in three interval); therefore, the corrected statistical significance was defined as *p* < 0.0083 (*p* < *p*_origin_/6). Box-and-whisker plots were performed to visually present the significant differences in the coupling strength between the two states for each group. The three horizontal lines on each box are the 25th, 50th, and 75th percentiles. The lines above and below each box represent the highest and the lowest values, respectively.

## 3. Results

### 3.1. Systemic Measurements

**Figure 3:**
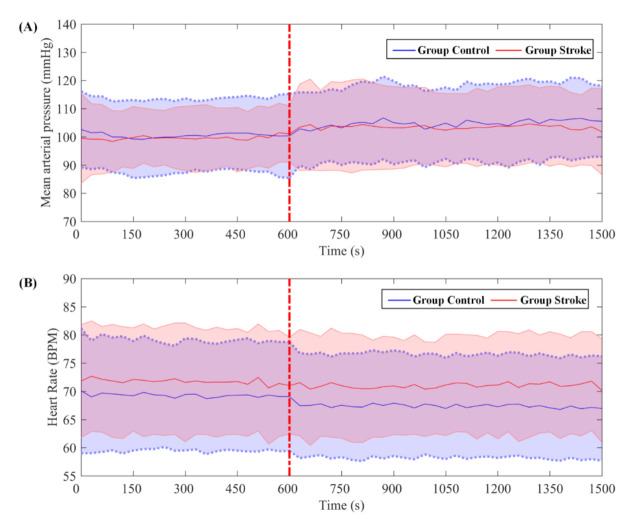
Group-averaged changes measured at State_R and State_ISPC in (**A**) mean arterial pressure and (**B**) heart rate, respectively. The red dotted line marks the start of the ISPC intervention. The middle solid line represents the mean of the signal, and the shaded area shows 95% confidence intervals.

A comparison between the systemic measurement parameters of the two states is shown in Figure 3. During the ISPC intervention, the mean arterial pressure increased and the heart rate decreased in both groups, although no significant differences were observed.

### 3.2. Cerebral Oscillation Measurements

**Figure 4:**
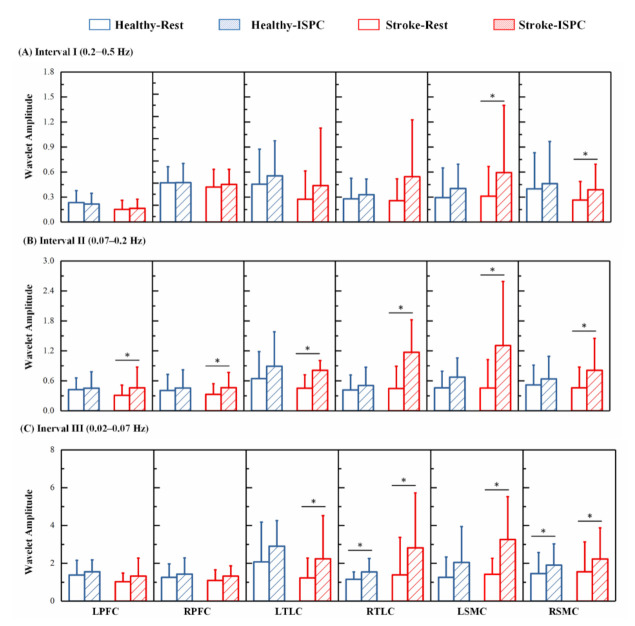
Comparison of the mean wavelet amplitudes of Δ [O_2_Hb] signals and the associated standard deviations for each group in each state is shown in (**A**) interval I, (**B**) interval II, and (**C**) interval III. Significant differences are marked with ∗ (*p* < 0.0083).

A comparison of the group-averaged wavelet amplitudes for each state in the three frequency bands is shown in Figure 4. The results showed that in interval I, the wavelet amplitude of the Δ [O_2_Hb] signals increased following ISPC intervention in the LSMC and RSMC (*p* < 0.0083) in patients with cerebral infarction. The wavelet amplitude was significantly increased in the bilateral RFC, TLC, and SMC (*p* < 0.0083) in interval II in the cerebral infarction patients. Additionally, the wavelet amplitude was significantly increased in the bilateral TLC and SMC (*p* < 0.0083) in interval II in patients with cerebral infarction. For the healthy controls, the wavelet amplitude of Δ [O_2_Hb] was higher in State_ISPC than in State_R in each interval and significantly higher in RTLC (*p* = 0.0047) and RSMC (*p* = 0.0013) in interval III.

### 3.3. Relationship between the Cerebral and Systemic Oscillations

For each participant, coupling interactions between ABP and Δ [O_2_Hb] signals of all 24 channels in different states and frequency intervals were computed. To more clearly characterize the coupling between ABP and Δ [O_2_Hb], channel-wise coupling strength values were averaged in six regions of interest. The region-wise coupling strength mutually directed interactions between the ABP and Δ [O_2_Hb] of the six regions were as follows: ABP → LPFC, ABP → RPFC, ABP → LSMC, ABP → RSMC, ABP → LTLC, and ABP → RTLC (oscillation 1 → oscillation 2 indicates that oscillation 1 influenced on oscillation 2). The coupling strength quantifies how the amplitudes of the oscillations in ABP are transmitted to the oscillations of Δ [O_2_Hb].

Repeated-measures ANOVA on the frequency-specific coupling parameters with the between-subject factor “group” and the within-subject factor “condition” revealed that the coupling strength values in interval I showed significant main effects of condition in the following coupling: ABP → LTLC (*p* = 0.015) and ABP → RSMC (*p* = 0.007). Additionally, the coupling strength in interval I showed a significant main effect of group in the ABP→RSMC coupling (*p* = 0.022). The coupling strength in intervals II and III showed significant main effects of condition in the ABP → LPFC, ABP → RPFC, ABP → LTLC, ABP → RTLC, ABP → LSMC, and ABP → RSMC couplings (*p* < 0.05). In interval II, a significant two-way interaction (*p* < 0.05) existed between group and condition in the ABP → RPFC, ABP → LTLC, and ABP → RTLC couplings. No significant effects of group × condition interactions were found in any interval.

### 3.4. Changes in the Coupling Strength

**Figure 5:**
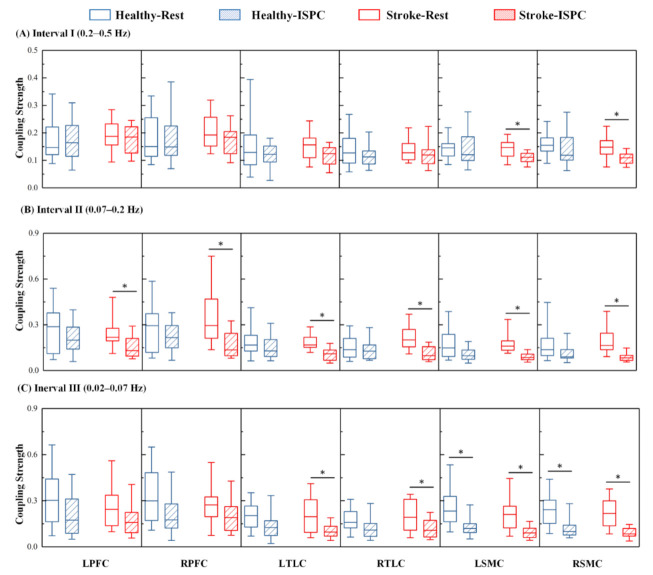
Comparison of region-wise coupling strength and the associated standard deviations from ABP to Δ [O_2_Hb] signals for each group in each state is shown in (**A**) interval I, (**B**) interval II, and (**C**) interval III. Error bars represent the standard deviations of the measurements. Significant differences are marked with ∗ (*p* < 0.0083).

The comparison of the group-averaged coupling strengths of Control and Stroke groups during the resting and ISPC states in the three frequency bands is shown in Figure 5, and the significance values are summarized in Table 3. The results showed that, for healthy controls, the coupling strength from ABP to Δ [O_2_Hb] was significantly lower in State_ISPC than in State_R in the bilateral SMC in interval III. For the cerebral infarction patients, the coupling strength from ABP to Δ [O_2_Hb] oscillations were significantly lower in the LSMC and RSMC during State_ISPC in interval I. In interval II, the coupling strength from ABP to Δ [O_2_Hb] was significantly lower in State_ISPC than in State_R in the bilateral PFC, TLC, and SMC in patients with cerebral infarction. Additionally, the coupling strength from ABP to Δ [O_2_Hb] was significantly decreased in State_ISPC than in State_R in bilateral TLC and SMC in patients with cerebral infarction in interval III.

## 4. Discussion

In this study, network coupling between Δ [O_2_Hb] and ABP was employed to evaluate the effect of the ISPC intervention on brain–cardiovascular interactions in poststroke subjects. The results show that following the ISPC intervention, the coupling strength from ABP to Δ [O_2_Hb] oscillations was significantly lower in interval II in the bilateral PFC, SMC, and TLC in cerebral infarction patients. Additionally, the coupling strength from ABP to Δ [O_2_Hb] oscillations was significantly lower in intervals III in the bilateral SMC in both groups. This phenomenon indicated that ISPC intervention might alter effective coupling interactions between ABP and cerebral hemodynamics.

In accordance with a previous study, no significant differences were found in mean arterial pressure or heart rate during ISPC intervention [32]. Although no significant differences were found, the mean arterial pressure was higher during ISPC intervention in both groups. This may be attributable to a higher resistance and rigidity of the vascular bed caused by atherosclerosis or age, resulting in weakening of the buffering effect on blood pressure. The decrease in heart rate might be caused by the baroreflex-mediated response to the increase in mean arterial pressure [33].

The fNIRS spectra contain information on both passive components such as heart and breathing pulses and active components such as spontaneous oscillation in the low-frequency oscillation range (i.e., interval II, 0.07–0.20 Hz; interval III, 0.02–0.07 Hz) [34]. Specific frequency bands in optical spectra are reportedly linked to cerebral autoregulation as a protective mechanism to maintain constant cerebral blood flow. Cerebral autoregulation acts as a high-pass filter, in which slower fluctuations that may result in a greater sustained impact on neurophysiological health are effectively buffered, whereas the faster, transient fluctuations in ABP are transmitted to the cerebral circulation almost linearly [35]. The frequency intervals II and III used in the present work incorporate the frequency ranges where cerebral autoregulation is considered to be operant. The spontaneous oscillations in interval II are associated with vasomotion and sympathetic activity, whereas the spontaneous oscillations in interval III are associated with neural network activity [4,36]. In this study, the lower coupling strength in intervals II and III suggests that ABP oscillations have a weaker influence on Δ [O_2_Hb] fluctuations. That is, fluctuations in ABP are weakly transmitted to the Δ [O_2_Hb] signal, which indicates a reduced transmission of pulsatile energy to the peripheral vessels, and therefore representing better cerebral autoregulation. The significantly higher wavelet amplitude in patients with cerebral infarction in interval II and interval III suggests that the ISPC intervention resulted in a significant hemodynamic response, which might be related to cerebral autoregulation.

The interval II cerebral hemodynamic changes may be affected by systemic control of the blood circulation [37]. Group × condition interaction was found to have a significant effect in interval II, which indicates that the effects of ISPC intervention on cerebral microcirculation differ in patients with cerebral infarction and elderly healthy individuals. Cerebral autoregulation ensures the stability of cerebral blood flow under fluctuations in cerebral perfusion pressure. In this interval, the coupling strength between State_R and State_ISPC was not significantly different in Group Control, suggesting that cerebral blood flow remained stable in the healthy subjects. This may be the result of the healthy participants being sustained by the cerebral autoregulation mechanism. However, the coupling strength was significantly lower in the bilateral PFC, TLC, and SMC in Group Stroke in this interval, which indicates that ISPC intervention facilitates cerebral microcirculation in the above cerebral regions in patients with cerebral infarction. Impaired autoregulation after stroke is hypothesized to be the result of damage to small cerebral arteries and capillaries caused by chronic diseases such as hypertension [38,39]. ISPC intervention augments cerebral blood flow in cerebral infarction patients, possibly through impaired cerebral autoregulation. A study has shown that increased global cerebral blood perfusion may promote the metabolism of neuronal and glial cells throughout the brain [40], which might contribute to the plasticity of the cerebral cortex system and assist recovery in patients with cerebral infarction.

The coupling strength from ABP to Δ [O_2_Hb] was significantly lower in both groups in interval III, which suggests that the physiological mechanism of ISPC for improving cerebral autoregulation function might be related to neurogenic regulation. The autonomous nervous system participates in vasoconstriction by regulating the release of substances that affect the activities of smooth muscles [41]. The rapid increase in blood flow velocity induced by ISPC produces strong shear stress on the vascular endothelium, which induces the release of nitric oxide or related compounds and thereby causes systemic vasodilation [42,43]. Consequently, the capacity of the cerebrovascular to buffer against dynamic pressure fluctuations is enhanced. The SMC plays an important role in sensation and motor control [44,45]. This study found that the coupling strength decreased significantly in the SMC area, suggesting that ISPC interventions can have a positive impact on the cerebral autoregulation function of the SMC. This result appears to imply that ISPC intervention may promote motor function recovery through its positive influences on motor-related networks.

The high frequency range (interval I, 0.20–0.50 Hz) was related to the effects of respiratory activities [26,46]. For the cerebral infarction patients, the coupling strength from ABP to Δ [O_2_Hb] oscillations was significantly lower in State_ISPC than in State_R in interval I, which indicates that the response of the respiratory activity to ABP fluctuations following ISPC intervention was weakened. This may be due to the less stable cardiorespiratory coupling in cerebral infarction patients [47,48]. This result may be an indication of impaired regulation of respiration observed via the cerebral hemodynamic signal. The decreased coupling strength was mainly distributed in SMC, which was probably related to the impairment of motor regions in patients with cerebral infarction.

The reduced coupling strength in State_ISPC suggests that IPSC rehabilitation intervention has a beneficial effect on the cerebral autoregulation state in poststroke patients. However, contrary outcomes were observed in a subset of patients. This suggests that IPSC (0.03 Hz) rehabilitation intervention improves the cardio-cerebral coupling state in most patients, whereas negative effects may occur in some patients. We speculate that this may be because ISPC rehabilitation does not apply to certain patients. Unfortunately, no regularity was discovered from the medical records of those patients. Another possible speculation is that the negative impact of ISPC on cerebral autoregulation may be random. This phenomenon may be attributed to the inability of the ISPC device to accurately synchronize with the cardiac cycle. ISPC intervention results in periodic alternation between a low oscillatory/high net shear profile during cuff deflation and a high oscillatory/low net shear rate pattern during cuff inflation [43]. This periodic opposing shear stimulus may be counteracted by the fact that the ISPC device is not synchronized with the cardiac cycle, which results in no net effect on vascular function, or even the opposite effect. Nevertheless, further follow-up with a large sample is needed to confirm this.

In recent years, as an ISPC device, the application of enhanced external counter pulsation has attracted more and more attention. Compared with ISPC devices, the cuffs of enhanced external counterpulsation inflate sequentially from distal to proximal during diastole and release pressure before the start of systole; thus, its sequenced activity is precisely synchronized with the cardiac cycle [49]. This equipment has been shown to improve the perfusion of vital organs and is beneficial for the recovery of cerebral blood flow in ischemic stroke patients [50,51]. The results of this study might be further understood by comparing the effects on cerebral autoregulation function under ISPC intervention and enhanced external counterpulsation intervention.

## 5. Limitations

Firstly, in this study, the gender ratio was heavily skewed toward males. However, the main results of this study would not be affected by the imbalanced sex distribution, as the most important results are based on intraindividual differences between conditions. In the future, additional female participants need to be recruited to improve understanding of the coupling between ABP and cerebral oxyhaemoglobin. Secondly, only ischemic stroke was studied in this study. Because of the pathophysiological differences between ischemic and haemorrhagic stroke, more participants with different types of strokes therefore need to be recruited in the future to enhance the understanding of physiological mechanisms of ISPC intervention.

## 6. Conclusions

In conclusion, the lower coupling strength from ABP to Δ [O_2_Hb] in the bilateral PFC, SMC, and TLC in interval II in State_ISPC indicated that ISPC intervention facilitates cerebral circulation in those cerebral regions in patients with cerebral infarction. The lower coupling strength in interval III in the SMC indicates that ISPC intervention may be promoted to motor function recovery through positive influences on motor-related networks. The obtained results on the coupling between Δ [O_2_Hb] and ABP offer new insights into the potential therapeutic effect of ISPC intervention on cerebral hemodynamic and guide therapy in clinical situations.

## Figures and Tables

**Table 1:** Comparison of demographic characteristics between the two groups.

Characteristic	Group Control	Group Stroke	*p* for Difference
**Age (years old)**	59.8 ± 5.9	63.9 ± 12.5	0.188
**Body Mass Index**	24.8 ± 3.1	25.8 ± 3.4	0.311
**Female Sex (%)**	10%	13.6%	0.724

Values are presented as mean ± standard error and percentages.

**Table 2:** Clinical characteristic of patients with cerebral infarction.

NO.	Gender	Age (years old)	Hemiplegia Side	Post Stroke (month)	Lesion Location	Other Diseases
**1**	M	72	L	3	R cerebellum	HT, HLP
**2**	F	81	R	2.5	L basal ganglia, frontal lobe	HLP
**3**	F	67	L	1	R basilar artery	HT, HLP
**4**	M	74	R	1.5	L basal ganglia	HT, HLP
**5**	M	77	R	3	L ventricle, thalamus	HT, DM
**6**	M	49	R	4	L basal ganglia	HT, HLP
**7**	M	61	R	1.5	L pons	HT, DM, HLP
**8**	M	74	R	3	L basal ganglia	HT, HLP
**9**	M	61	R	2.5	L pons	HT, DM, HLP
**10**	M	46	L	1.5	R basal ganglia	/
**11**	M	55	L	2	R pons	HT, HLP
**12**	M	61	R	1.5	L thalamus	HT, DM, HLP
**13**	M	74	R	4	L basal ganglia	HT, HLP
**14**	M	46	L	2.5	R basal ganglia	/
**15**	M	82	L	6	R brainstem	DM, HLP
**16**	M	46	L	3.5	R basal ganglia	/
**17**	M	61	R	1.5	L thalamus	HT, DM, HLP
**18**	M	74	R	3	L temporal lobe, parietal lobe	HT, HLP
**19**	M	82	L	6	R brainstem	DM, HLP
**20**	F	48	R	1	L frontal lobe, insular cortex, basal ganglia	HT, DM, HLP
**21**	M	61	R	6	L thalamus	HT, HLP
**22**	M	53	R	3	L basal ganglia, pons, thalamus, temporal lobe	HT

M, male; F, female; L, left; R, right; HT, hypertension; DM, diabetes mellitus; HLP, hyperlipidemia.

**Table 3:** Statistical significance results of the average differences of coupling strength values between the State_R and State_ISPC.

	Interval I		Interval II		Interval III	
	Group	*p* value	Group	*p* value	Group	*p* value
**ABP** **→** **LPFC**	Group Stroke	0.454	Group Stroke	0.001	Group Stroke	0.033
	Group Healthy	0.705	Group Healthy	0.319	Group Healthy	0.112
**ABP** **→** **RPFC**	Group Stroke	0.114	Group Stroke	< 0.001	Group Stroke	0.097
	Group Healthy	0.653	Group Healthy	0.227	Group Healthy	0.085
**ABP** **→** **LTLC**	Group Stroke	0.009	Group Stroke	< 0.001	Group Stroke	< 0.001
	Group Healthy	0.213	Group Healthy	0.343	Group Healthy	0.022
**ABP** **→** **RTLC**	Group Stroke	0.289	Group Stroke	< 0.001	Group Stroke	0.006
	Group Healthy	0.290	Group Healthy	0.238	Group Healthy	0.039
**ABP** **→** **LSMC**	Group Stroke	< 0.001	Group Stroke	< 0.001	Group Stroke	< 0.001
	Group Healthy	0.803	Group Healthy	0.028	Group Healthy	0.001
**ABP** **→** **RSMC**	Group Stroke	< 0.001	Group Stroke	< 0.001	Group Stroke	< 0.001
	Group Healthy	0.400	Group Healthy	0.019	Group Healthy	< 0.001

*p* values for differences are calculated using one-way ANOVA.

## Data Availability

The data presented in this study are available upon request from the corresponding author.
